# Assessing Movement Factors in Upper Limb Kinematics Decoding from EEG Signals

**DOI:** 10.1371/journal.pone.0128456

**Published:** 2015-05-28

**Authors:** Andrés Úbeda, Enrique Hortal, Eduardo Iáñez, Carlos Perez-Vidal, Jose M. Azorín

**Affiliations:** Brain-Machine Interface Systems Lab, Miguel Hernández University, Av. de la Universidad S/N, 03202 Elche, Spain; University of Chicago, UNITED STATES

## Abstract

The past decades have seen the rapid development of upper limb kinematics decoding techniques by performing intracortical recordings of brain signals. However, the use of non-invasive approaches to perform similar decoding procedures is still in its early stages. Recent studies show that there is a correlation between electroencephalographic (EEG) signals and hand-reaching kinematic parameters. From these studies, it could be concluded that the accuracy of upper limb kinematics decoding depends, at least partially, on the characteristics of the performed movement. In this paper, we have studied upper limb movements with different speeds and trajectories in a controlled environment to analyze the influence of movement variability in the decoding performance. To that end, low frequency components of the EEG signals have been decoded with linear models to obtain the position of the volunteer’s hand during performed trajectories grasping the end effector of a planar manipulandum. The results confirm that it is possible to obtain kinematic information from low frequency EEG signals and show that decoding performance is significantly influenced by movement variability and tracking accuracy as continuous and slower movements improve the accuracy of the decoder. This is a key factor that should be taken into account in future experimental designs.

## Introduction

Brain-Machine Interfaces (BMIs) provide a new hope to restore motor functions of the severely disabled people by controlling external devices with volitional commands extracted from brain signals [1–3]. For this reason, the use of BMIs as an assistive technology for motor substitution has been widely explored [4, 5]. The recent advances in BMI technology allow training patients to control their brain signals and enable people with severe motor disabilities to interact with their environment by bypassing their impaired neuromuscular system [6–8]. In the case of stroke, motor impairment is the major cause of permanent disability. These people usually suffer from upper limb movement limitations in their affected side, and the recovery of the arm movement is often variable and incomplete [9]. This recovery is crucial in order to perform activities of their daily life, so the assistance during the rehabilitation may be a key factor of improvement [10]. In these sense, BMIs could be a very useful tool to induce plasticity in motor recovery procedures [11].

A rapid development of upper limb kinematic decoding techniques has arisen from intracortical recordings of brain signals during the last decades. Now, it is possible to decode information obtained from a large population of neurons, or even a single neuron, into accurate kinematic parameters [12, 13]. In some studies, the motor cortical activity of monkeys was used to perform reaching and grasping activities with a robotic arm [14], or to perform three dimensional movements that included grasping for self-feeding using a mechanical device [15]. Invasive approaches have been successfully used in people with motor disabilities to perform reaching and grasping tasks [16, 17]. So far, the remarkable progress of this approach has provided a solid step towards the feasibility of the application of intracortical decoding not only under laboratory conditions, but also in a real world environment. Thus, the use of these techniques can be very useful to control robotic exoskeletons.

However, the use of non-invasive approaches to perform similar decoding procedures is still in its early stages. Currently, a direct neural control from non-invasive brain recordings is far from being achieved. Recent offline studies show that there is a correlation between electroencephalographic (EEG) signals and hand-reaching kinematic parameters [18, 19]. This correlation was found after applying a simple decoder based on linear regression between signals. In these works, the volunteers were asked to perform random selections of eight targets in a 3D environment. The results showed that better correlations between velocity and EEG recordings were found when the users performed linear hand-reaching movements and decreased when the movement variability was higher. In other works, the use of low frequency components (< 2Hz) showed high correlation results when performing continuous right arm movements [20]. In this case, volunteers were asked to perform natural arm movements with a variable speed. The existence of an actual relationship between EEG signals and upper limb kinematics is really encouraging, but there is still a great gap between current findings and an accurate and reliable non-invasive neural decoding. Linear decoding methods applied to low frequency EEG signals are rather controversial and there is no general agreement about the metrics used to compare decoded and real kinematics. In [21], authors claim that there is a misinterpretation of the results obtained from offline approaches [18]. Nonetheless, first closed loop attempts have already been undertaken in recent studies by using a discrete four target visual interface [22]. Again, there is a current discussion about the methods employed to assess performance, which may throw overoptimistic results similar to the ones obtained from random data [23, 24].

Here, we assess the validity of linear decoders in tracking activities using a manipulandum. On this basis, what seems to be clear is that the accuracy of upper limb kinematics decoding depends partially on the characteristics of the performed movement in terms of velocity, trajectory and variability. As a consequence, different arm movements have been evaluated to quantify the influence of speed and movement variability in the decoding performance. The results confirm that it is possible to obtain kinematic information from low frequency EEG signals and show that decoding performance is significantly influenced by movement variability and tracking accuracy as slower and more continuous movements obtained a better correlation. This is a key factor that should be taken into account in future experimental designs.

## Material and Methods

### Subjects

Five healthy volunteers (all male and right-handed) with ages between 25 and 30 years (mean 27.8±2.0) took part in the experiments. All volunteers are engineering students or researchers and are familiar to the technologies applied in this work. EEG human recordings used in this study have been approved by the ethics committee of the Miguel Hernández University of Elche, Spain. Written consent according to the Helsinki declaration was obtained from each subject.

### Experimental Paradigm

**Fig 1:**
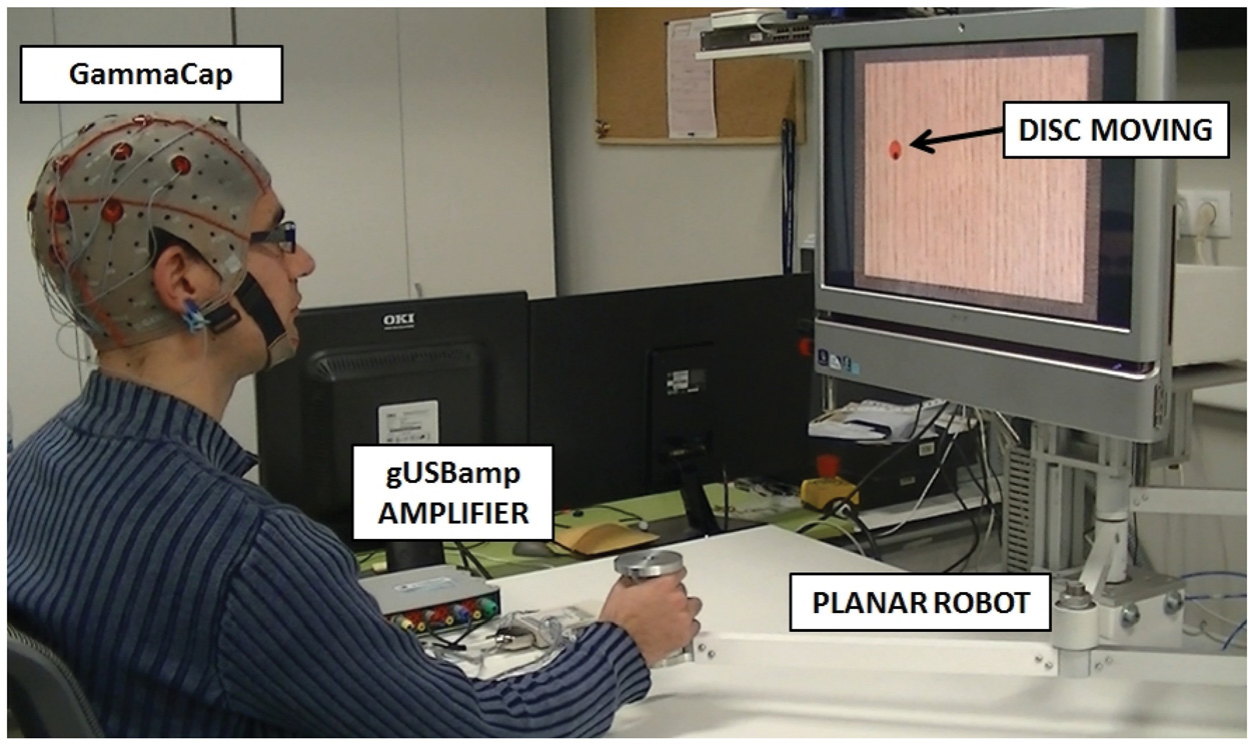
Experimental environment showing the subject performing the tracking movements in front of a screen. The subject should follow the red circle by controlling a black cursor with the planar manipulandum.

**Fig 2:**
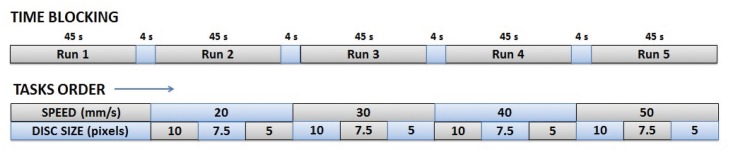
Blocking structure of the experimental procedure. Time blocking (up) and tasks order (down).

The volunteers were asked to follow a disc that moved randomly on the screen with a constant speed by controlling a black cursor (Fig 1). To that end, the disc randomly changed its orientation each 100 ms (10 degrees clockwise or anti-clockwise) and instantaneously moved forward a particular amount of pixels depending on the speed of the disc. In previous works, it was proved that subjects do not fixate on the moving object but on the initial and final point of the movement [25]. However, in these tests, subjects were specifically asked to focus on the cursor movement without losing sight of the tracked disc. This condition was aimed at preventing the appearance of ocular artifacts due to fast ocular movements back and forth to localize the disc. To control the cursor, the volunteer had to move the end effector of a planar manipulandum inside a workspace of 225 × 150 mm (1350 × 900 pixels). Four different speeds were defined (20, 30, 40 and 50 mm/second). For each speed, 3 sessions, with a different disc size (5, 7.5 and 10 pixels/diameter), were performed. Each session consisted of five runs of continuous movements during 45 seconds. After each run, a success percentage, representing the time the volunteer was able to stay inside the disc, was shown and a resting period of 4 seconds was included between runs (for more details, see Fig 2). The total time of the experiment was about 1 hour. All volunteers sat in front of a computer screen in an isolated room to avoid disturbances during the recordings.

### EEG Recordings and Preprocessing

For the recordings, a g.GAMMACap with 16 sintered Ag/AgCl ring electrodes g.LADYbird (gTec, GmbH, Austria) was used. The electrodes were placed over the scalp with the following distribution: FC5, FC1, FC2, FC6, C3, Cz, C4, CP5, CP1, CP2, CP6, P3, Pz, P4, PO3 and PO4, according to the International 10/10 System. The electrodes placement was chosen around motor and premotor cortex which is the area where more information was expected to appear. Moreover, frontal electrodes, which can be influenced by ocular artifacts, were not selected. EEG signals were registered and amplified through a g.USBamp device (gTec, GmbH, Austria) with a sampling rate of 1200 Hz and then resampled to 120 Hz. EEG signals were also band-pass filtered between 0.1 and 200 Hz with a high-order Chebyshev filter. Then, a zero-phase 4th order Butterworth low-pass filter was applied to eliminate frequencies higher than 2 Hz. Finally, EEG data from each electrode were standardized by subtracting, for each time sample (*t*), the mean ($\bar{V}$) of the signal and dividing the result by the standard deviation (*SD*_*V*_) as shown in Eq (1). This standardization was computed for each performed run and prior to the decoding procedure.

$$\begin{array}{c} EV\left[t\right]=\frac{V\left[t\right]-\bar{V}}{SD_{V}} \end{array}$$ (1)

### Data Decoding Procedure

To decode the position of the upper limb, a similar procedure to the already used to decode hand velocity in [18] has been applied. In Eq (2) and Eq (3), the linear model used to decode hand position from EEG signals is shown. The transformation parameters *a* and *b* represent weight variables obtained from a linear regression. In Eq (2), *x*[*t*] represents the position of the hand in the X-Axis at time *t*. In Eq (3), *y*[*t*] represents the position of the hand in the Y-Axis at time *t*. For both equations, *N* represents the total number of electrodes (16 in this case) and *L* (with a value of 10) is the number of time lags. A gap of 10 time samples between each selected lag has been introduced to match the processing window to approximately 83 ms. This processing time window was selected similar to previous studies [18]. *S*_*n*_[*t*−*k*] represents the voltage difference measured at sensor *n* in the lag time *k*.

$$\begin{array}{c} x[t]=a_{x}+\sum_{n=1}^{N}\sum_{k=0}^{L}b_{nkx}S_{n}[t-k] \end{array}$$ (2)

$$\begin{array}{c} y[t]=a_{y}+\sum_{n=1}^{N}\sum_{k=0}^{L}b_{nky}S_{n}[t-k] \end{array}$$ (3)

The resulting decoded hand positions (*x*[*t*] and *y*[*t*]) have been compared to the original hand positions. To that end, the Pearson correlation coefficient has been obtained for each axis after performing a 5-fold cross validation after concatenating all five runs for each session and using one run as test data and the remaining four as model data. The average decoding performance (DP) is obtained for each speed and size of the disc. Additionally, shuffled data have been used as input to assess if the decoding accuracy was above chance levels. Shuffled data have been obtained by randomly mixing time points of the recorded data and then computed the same way as the original data. To that end, shuffled data have been filtered and standardized before computing decoding performance (as shown in EEG Recordings and Preprocessing section). Decoding coefficients have been computed 100 times to avoid chance effects due to the stochastic nature of the process.

### Movement variability

To compute the influence of the movement in the decoding performance, movement variability (MV) and tracking accuracy (TA) have been compared. This relationship has been obtained by computing the correlation coefficient (*r*) between all the values obtained for each speed and disc size. The *r* value has been averaged (mean±STD) between all subjects. To compute MV, the standard deviation of the position profiles (X and Y trajectories) has been obtained for each speed and disc size (averaged between runs). TA corresponds, for each condition, to the relative time the subject is able to correctly maintain the cursor inside the tracked disc for each run *i*, averaged between *N* runs, as shown in Eq (4).

$$\begin{array}{c} TA=\frac{\sum_{i}^{N}\frac{SuccessTime_{i}}{TotalTime_{i}}}{N} \end{array}$$ (4)

Additionally, the correlation of both parameters (MV and TA) with the decoding performance (DP) has been computed in a similar way.

### Electrodes and frequency contribution

The relative contribution of the recorded electrodes used to decode hand positions has been obtained for each time lag and electrode according to the formula:

$$\begin{array}{c} R_{n}=\sqrt{b_{nx}^{2}+b_{ny}^{2}} \end{array}$$ (5)

where *b*_*nx*_ and *b*_*ny*_ are the weight variables of the linear regression corresponding to each electrode *n*. The average across all folds has been computed and represented for each time lag through the topoplot function (EEGLAB toolbox of Matlab). To examine the importance of each time lag to the decoding, the relative contribution of each time lag was defined as follows:

$$\begin{array}{c} \%C_{i}=100\times \frac{\sum_{n=1}^{N}\sqrt{b_{nix}^{2}+b_{niy}^{2}}}{\sum_{n=1}^{N}\sum_{k=0}^{L}\sqrt{b_{nkx}^{2}+b_{nky}^{2}}} \end{array}$$ (6)

for time lags *i* from 0 to L, where %*Ci* is the relative contribution for a scalp map at time lag *i*.

Additionally, the Power Spectral Density (PSD) of the position profiles has been computed between 0.1 and 100 Hz and then, the power of the 0.1–2 Hz band has been divided by the power of the 0.1–100 Hz band to obtain the spectral contribution (SC) of low frequency components. These values have been compared with the decoding performance by computing the correlation coefficient (r) between all the values obtained for each condition.

## Results

**Fig 3:**
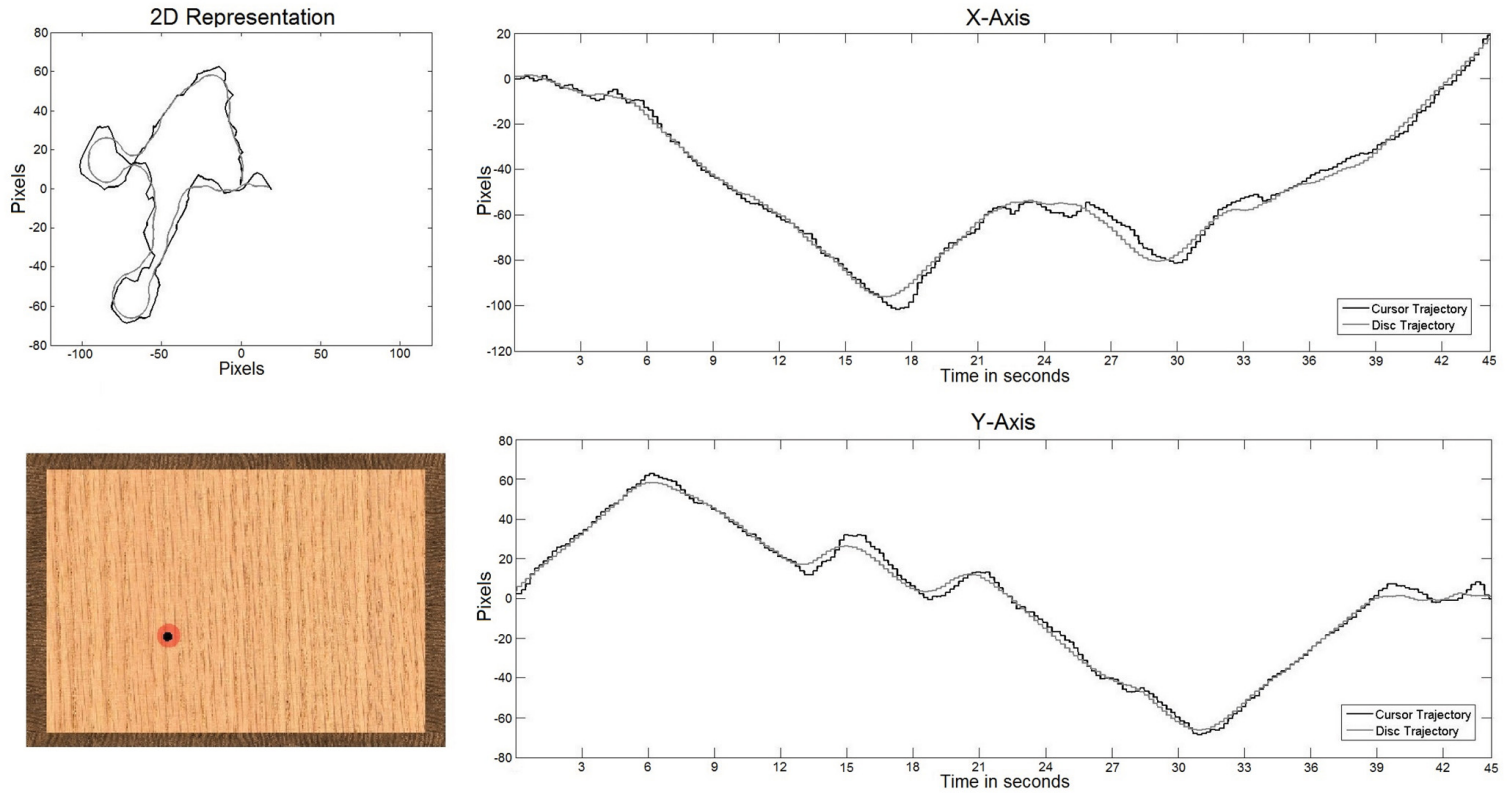
Example of the trajectory followed by the subject. 2D representation of the trajectory (top-left). X and Y axis trajectories (right). Appearance of the visual interface (bottom-left).

**Fig 4:**
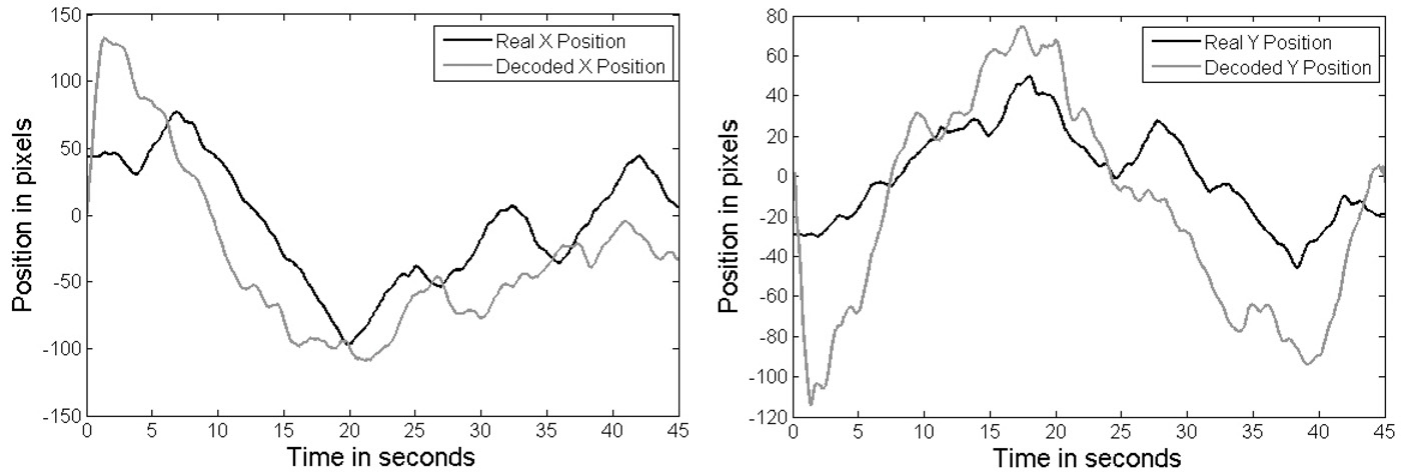
Example of a decoded trajectory for Subject 4. Horizontal axis (left) and vertical axis (right).

In Fig 3, an example of a bidimensional trajectory is shown (up-left). The gray path represents the original disc trajectory, while the black path shows the one performed by the subject controlling the planar manipulandum. Table 1 shows the tracking accuracy measured during the performed trajectory. This percentage represents the amount of time the volunteer is able to stay inside the disc during each run. The average tracking accuracy (*μ*) for each speed and disc size and the standard deviation (*STD*) is also presented. In Fig 4, an example of decoded trajectories is shown. The decoded *x* and *y* position is shown for Subject 4 with the minimum speed (20 mm/s) and the smallest disc size. The decoded trajectory (gray) follows the original trajectory (black).

**Table 1:** Accuracy percentages (%) for different disc speeds (mm/s) and sizes (diameter in pixels).

SPEED	SIZE	S1	S2	S3	S4	S5	AVG
20	10	99.29	99.56	97.69	99.47	87.38	96.68±5.25
	7.5	96.71	96.89	96.62	97.69	73.29	92.24±10.60
	5	93.24	82.22	84.53	87.29	42.44	77.94±20.27
30	10	96.00	97.87	95.38	94.67	63.24	89.43±14.69
	7.5	90.76	84.71	88.89	88.89	50.27	80.70±17.15
	5	73.78	59.56	70.93	68.98	27.51	60.15±19.01
40	10	28.31	45.64	38.31	37.11	45.91	39.59±6.29
	7.5	22.36	16.18	33.38	25.42	37.47	26.96±8.53
	5	16.00	6.09	15.38	9.38	15.91	12.55±4.55
50	10	28.22	27.11	35.11	31.96	29.73	30.43±3.19
	7.5	21.33	13.29	24.00	15.64	17.64	18.38±4.31
	5	9.60	4.22	12.36	6.13	9.24	8.31±3.18

**Fig 5:**
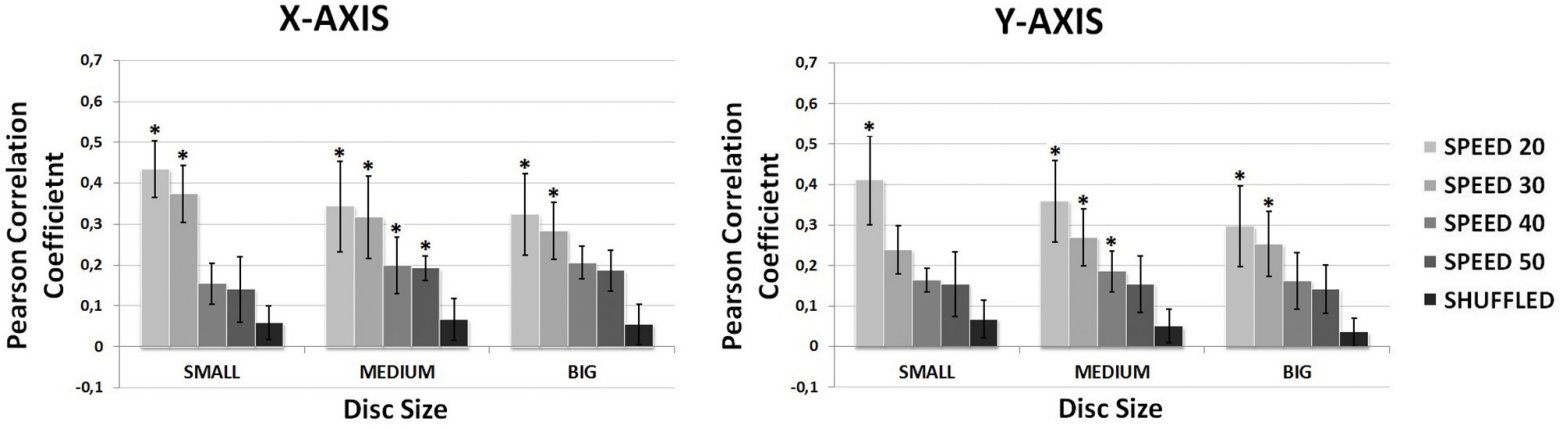
Decoding performance regarding speed (mm/s) and disc size (small, medium, big). The stars represent significant differences with respect shuffled condition.

Table 2 shows the Pearson correlation coefficients obtained for each volunteer after performing the hand position decoding. The results are shown for each speed and disc size regarding X-axis and Y-axis. The mean (*μ*) and standard deviation (*STD*) is also presented for each axis and speed, showing a decrease in the decoding performance for faster trajectories. Fig 5 shows a graphical representation of the averaged Pearson correlation coefficients for the different disc sizes and speeds. It also presents the results for shuffled condition. The significance of the decoding performances has been analyzed from these results. To that end, shuffled condition has been compared to the average decoding coefficients obtained for different disc sizes and speeds running a Wilcoxon Sum-Rank Test with a confidence interval of 90% and then applying a Bonferroni-Holm iterative correction for multiple comparisons. This analysis has shown that decoding performance of slow speeds (20 and 30 mm/s) is predominantly above chance levels (p < 0.1) for both axes and for all disc sizes. In the case of faster trajectories (40 and 50 mm/s), this occurs only for a medium disc size.

**Table 2:** Pearson correlation coefficients after decoding hand trajectories performed with the planar manipulandum for different disc speeds (mm/s) and sizes (diameter in pixels).

**SUBJECT**		**S1**		**S2**		**S3**	
SPEED	SIZE	X-AXIS	Y-AXIS	X-AXIS	Y-AXIS	X-AXIS	Y-AXIS
20	10	0.38	0.37	0.38	0.38	0.13	0.20
	7.5	0.34	0.40	0.22	0.39	0.41	0.22
	5	0.35	0.40	0.44	0.41	0.37	0.37
30	10	0.19	0.20	0.22	0.17	0.19	0.31
	7.5	0.38	0.25	0.30	0.20	0.49	0.29
	5	0.26	0.24	0.32	0.27	0.37	0.13
40	10	0.18	0.19	0.20	0.11	0.13	0.26
	7.5	0.17	0.27	0.15	0.15	0.15	0.20
	5	0.22	0.17	0.08	0.12	0.15	0.18
50	10	0.16	0.10	0.21	0.14	0.14	0.12
	7.5	0.15	0.11	0.21	0.18	0.17	0.16
	5	0.12	0.10	0.06	0.16	0.10	0.10
**SUBJECT**		**S4**		**S5**		**MEAN±STD**	
SPEED	SIZE	X-AXIS	Y-AXIS	X-AXIS	Y-AXIS	X-AXIS	Y-AXIS
20	10	0.29	0.29	0.30	0.14	0.30±0.10	0.27±0.10
	7.5	0.26	0.45	0.13	0.25	0.27±0.11	0.34±0.10
	5	0.34	0.54	0.25	0.23	0.35±0.07	0.39±0.11
30	10	0.36	0.29	0.22	0.14	0.24±0.07	0.22±0.08
	7.5	0.28	0.35	0.23	0.19	0.34±0.10	0.25±0.07
	5	0.43	0.29	0.27	0.26	0.33±0.07	0.24±0.06
40	10	0.16	0.09	0.10	0.10	0.15±0.04	0.15±0.07
	7.5	0.29	0.19	0.12	0.16	0.18±0.07	0.19±0.05
	5	0.14	0.19	0.16	0.22	0.15±0.05	0.17±0.03
50	10	0.23	0.17	0.26	0.25	0.20±0.05	0.16±0.06
	7.5	0.23	0.15	0.20	0.29	0.20±0.03	0.18±0.07
	5	0.26	0.28	0.14	0.22	0.13±0.08	0.17±0.08

**Fig 6:**
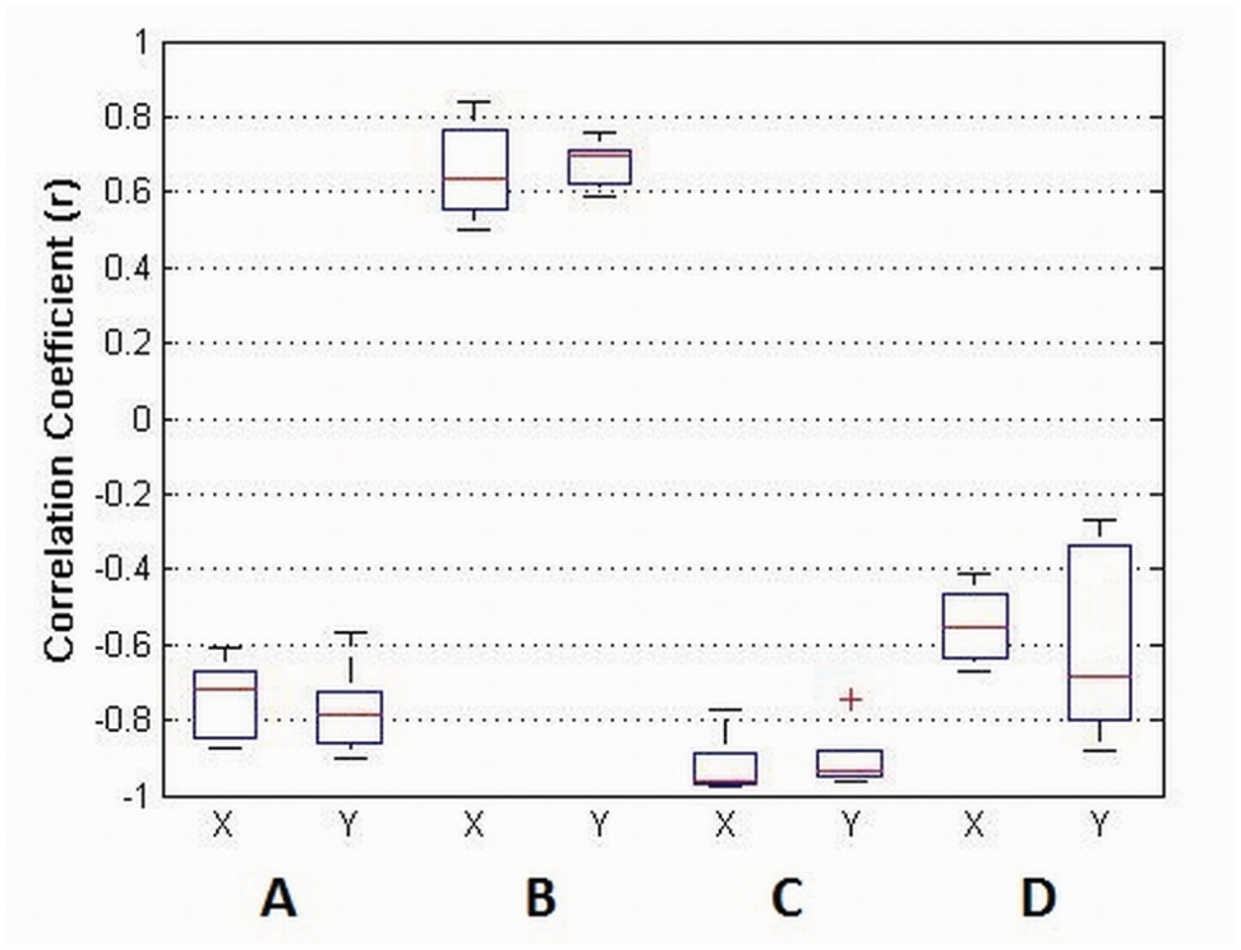
*r* values between different compared parameters: Movement Variability (MV), Decoding Performance (DP), Tracking Accuracy (TA) and Spectral Contribution (SC) of the position profiles for the 0.1–2 Hz band. A—MV vs DP, B—TA vs DP, C—MV vs TA and D—SC vs DP.

To obtain a proper relationship between movement factors, Movement Variability (MV) and Tracking Accuracy (TA) have been computed and compared between them and with Decoding Performance (DP) by calculating the correlation coefficient (r) averaged between subjects. The results show that MV has a high negative correlation with DP (Fig 6, A) and TA has a high positive correlation with DP (Fig 6, B). The correlation between TA and MV has also been calculated showing a high negative correlation (Fig 6, C). Additionally, a significant relationship between movement speed and TA was found after performing a non-parametric test (Kruskal-Wallis two-way ANOVA analysis) (p < 0.05) using DP as the dependant variable showing that higher speeds are correlated with a decrease in the decoding performance for both axis. The relative contribution of the 0.1–2 Hz band has been computed in relation to the contribution of the 0.1–100 Hz band for all the position profiles (each speed and disc size). The correlation between this spectral contribution (SC) and the decoding performance (DP) has been obtained showing high negative correlations (Fig 6, D).

## Discussion

The presence of significant decoding correlations in most of the experimental conditions differs from what is obtained in [21], where decoding performance of upper-limb movements was not above chance, and suggests that kinematic parameters of hand movement can be inferred from neural information through linear regression models. Nonetheless, the improvements obtained when reducing movement speed may be caused by the inherent properties of the correlation metric [21]. However, the decoding performance increases with lower contributions of the 0.1–2 Hz band suggesting that correlations are not caused (or at least not entirely caused) by the application of linear regression to similar frequency bands (both EEG slow cortical potentials and low frequency kinematics) and that there is an important influence of motor related cortical activations. Also, the decoding performance may be benefited by motor learning induced from a longer training with the manipulandum.

**Fig 7:**
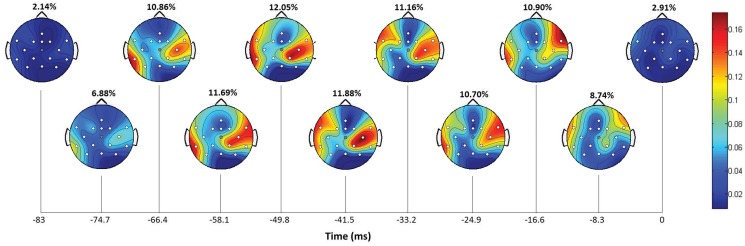
Contribution of the 16 recorded electrodes to the decoding performance for Subject 4, right arm (in gray, Cz electrode). The scalp maps are computed for each time lag (around 8.3 ms) and show parietal and strong central involvement. The relative contribution (%) for each lag is also computed showing a peak around 50 ms prior to the decoded instant corresponding to a 12.05% contribution. The remaining subjects show a similar behavior.

Fig 7 shows the individual contribution of the recorded electrodes to the decoding performance for the different time lags (Subject 4, right arm). The relative contribution of each time lag has also been computed. The scalp maps show a peak of contribution around 50 ms prior to the decoded instant corresponding to a 12.05%. The results show a high ipsilateral contribution of central regions and minor contributions of parietal regions, meaning that cortical activity is mainly centered on the motor cortex. This is similar to previous studies where activity, although mainly contralateral, revealed frontal, central and parietal involvement particularly on sensor CP3 [18] and it is more consistent with a recent study by the same authors [22]. In [20], this activity is distributed along the midline. The discrepancy of cortical areas involved in the decoding still needs a proper evaluation and it is an important issue for future research. However, all the studies agree to show an important contribution of the central area around 50 ms prior to the decoded instant.

Table 1 shows a natural decreasing in the TA when the speed increases and the disc gets smaller. This relationship is similar for most of the subjects. However, Subject 5 shows a very low TA compared to the rest of subjects likely biased by his/her ability to control the planar manipulandum. The high positive correlations between TA and DP suggest that the linear decoder behaves better in more accurate trajectories. This conclusion is also supported by the high negative correlation obtained between TA and MV, i.e., subjects had more difficulties to track the disc when the trajectory performed was more variable. Also, Fig 3 shows that slower speeds show a better decoding performance. This is not surprising as, in general, slower speeds decrease the difficulty of following the disc with the manipulandum and, as a consequence, are related to an increase in TA and a decrease in MV. These findings are consistent with the results obtained in [18], where movement variability was in inverse proportion to decoding performance. Also, the high correlation coefficients shown in [20], where natural and round hand reaching movements were assessed, point in the same direction.

## Conclusion

In this work, we reported the influence of speed, trajectory and movement variability in hand kinematics decoding by performing bidimensional trajectories with a planar manipulandum. To that end, five healthy volunteers were asked to follow a disc, which moved randomly on the screen with a constant speed, by controlling a cursor with the planar robot. The presence of significant decoding correlations in most of the experimental conditions suggests that kinematic parameters of hand movement can be inferred from neural information through linear regression models. The results also show that decoding performance (DP) is significantly influenced by movement variability (MV) and tracking accuracy (TA) as continuous and accurate movements obtained a better decoding performance. This is consistent with the results obtained in [18, 20], where continuous and linear movements obtained a high decoding performance. Additionally, the scalp maps represented for the recorded sensors show a high contribution of central regions and minor contributions of parietal regions, meaning that cortical activity is mainly centered on the premotor cortex.

The study has gone some way towards enhancing our understanding of the neural mechanisms during upper limb movement and it serves as a first step to apply this kinematics decoding technique to control assistive robotics in a more natural way. There is abundant room for further progress in determining how movement variability affect the decoding performance that could be aimed at particularizing experimental procedures to assess different movement factors. Also, in future works, real time testing is needed to show if this decoding is feasible in upper limb decoding applications. Also, brain signals variability of patients, particularly people suffering from a stroke, should be studied to prove the viability of this method in rehabilitation procedures. This research will serve as basis for future studies regarding upper limb kinematics decoding where further research to eliminate artifact influence and improve accuracy should be undertaken.
